# Laxative Effects of Yangyin Tongmi Capsule on a Model of Diphenoxylate-Induced Constipation in Mice

**DOI:** 10.1155/2020/1471824

**Published:** 2020-02-21

**Authors:** Shan Liu, Dayun Sui, Wenwen Fu, Xiaofeng Yu, Yuangeng Li, Xueji Wu, Yiping Hou, Minyu Guo, Huali Xu

**Affiliations:** Department of Pharmacology, School of Pharmaceutical Sciences, Jilin University, Changchun 130021, China

## Abstract

Constipation is characterized by reduced number of bowel movements, dry stools, and difficult defecation. Yangyin Tongmi capsule (YTC), a traditional Chinese formula, is used in the treatment of constipation, while the underlying mechanisms remain unknown. Herein, this work attempted to prove the effects of YTC on constipation treatment and its possible mechanisms. KM mice were randomly divided into four groups (*n* = 10/group) and treated with double distilled water (Control), diphenoxylate (Model: 10 mg/kg), or diphenoxylate plus low-dose YTC (L-YTC: 0.6 g/kg) or high-dose YTC (H-YTC: 1.2 g/kg). The data indicated that YTC can significantly shorten the discharge time of the first black stool, improve intestinal propulsion rate, and increase the water content and quantity of feces in mice. ELISA suggested that YTC regulate the content of intestinal hormones and neurotransmitters, such as motilin (MTL), gastrin (GT), somatostatin (SST), substance P (SP), acetylcholine (Ach), and nitric oxide (NO). The expression levels of aquaporin 3 (AQP3) and aquaporin 8 (AQP8) in the colon were examined by immunohistochemistry. In the meantime, the expression levels of P2X2, C-kit, and stem cell factor (SCF) in the colon were examined by western blot analysis. The results of this study suggest that YTC has mitigative effects on diphenoxylate-induced constipation by regulating the content of intestinal hormones and neurotransmitters and regulating the expression of related proteins in the colon.

## 1. Introduction

Constipation is a common digestive disease in clinical practice, and its characteristic manifestations are long-term persistent difficulty in defecation and incomplete defecation, which seriously affect the quality of life of most patients, and it also brings different degrees of pain and mental burden to people [1, 2]. Constipation can lead not only to gastrointestinal nerve dysfunction, but also cardiocerebrovascular disease and hepatic encephalopathy [3]. In recent years, with the rapid development of society, people's diet and lifestyle have also changed. Epidemiological surveys show that the incidence of constipation appears a clear upward trend [4, 5].

At present, the etiology and pathogenesis of constipation mainly include changes in intestinal neurotransmitters and gastrointestinal peptides, colonic dysfunction, and abnormal interstitial cells of Cajal (ICC) [6–11]. Constipation may be the result of those factors. The intestinal nervous system is of great significance for studying the pathogenesis of constipation. The intestinal nervous system has a relatively independent structure, it contains numerous ganglia and nerve fibers, and they intersect each other to form a reticular sulcus to control the gastrointestinal function [12]. The gastrointestinal tract produces a variety of gastrointestinal hormones, including excitatory transmitters represented by SP, Ach, and MTL and inhibitory transmitters represented by NO, GT, SST, etc. Various neurotransmitters play different effects on the enteric nervous system, coordinate and balance each other, and jointly complete the physiological functions of the intestine. The study showed that the levels of MTL and GT decreased in loperamide-induced constipation in Kunming mice, while NO increased [13]. Wattchow et al. [14] confirmed a decrease in the proportion of Ach in patients with slow transit constipation. SP can regulate the intestinal motility and secretory function through autocrine or paracrine action on the cell membrane surface, and increased SP expression can accelerate enteric nerve conduction [15]. Zhuang et al. [16] confirmed that kiwifruit fruit powder with high dietary fiber has a laxative effect on constipation mice by increasing SP content.

Aquaporins are expressed in the digestive system and play a physiological role, and their distribution is related to their functions. In the human colon, AQP3 and AQP8 are mainly expressed in mucosal epithelial cells [10, 17], which indicates their importance in the water transport effect. In addition, there are many research studies about ICC. ICC is a pacemaker of the gastrointestinal tract. It has the functions of producing and transmitting slow waves, regulating neurotransmitters, and so on [18, 19]. It is an important part of regulating gastrointestinal motility, and as an intermediary for controlling the movement of gastrointestinal smooth muscles in the enteric nervous system, ICC participates in the signal transduction of gastrointestinal neurotransmitters and the regulation of neurotransmitters [20]. Studies have shown that changes in the structure, morphology, or quantity of ICC in the intestine may be a necessary mechanism for constipation [21, 22]. The SCF/C-kit signaling pathway can affect the phenotype and function of ICC. Due to the reversibility of ICC phenotype, it provides an important target for the treatment of constipation [23].

Many natural plants such as *Malva species* have an effect on constipation [24]. Carla Cirillo [25] has reviewed the most common botanical laxatives such as *senna, cascara, frangula, aloe,* and *rhubarb* and their use in the treatment of constipation. In addition, the active ingredients in botanical products can also be used to alleviate constipation, such as *Rhein* extracted from *Cassia fistula* pod pulp is used as an active ingredient in laxatives [26]. Yan et al. [27] confirmed that aqueous extracts of *Herba Cistanche* promoted intestinal motility in loperamide-induced constipation rats by ameliorating the interstitial cells of Cajal. YTC is composed of *Herba Cistanches, Semen sesami nigrum, Radix angelicae sinensis, Semen armeniacae amarum, Semen persicae, Radix clematidis, Radix scrophulariae, Semen pruni*, *and Fructus aurantii*. However, there have been no reports on the effect of YTC in the development of constipation and its relevant mechanisms. Therefore, the current study was designed to explore the effect of YTC on diphenoxylate-induced constipation.

## 2. Materials and Methods

### 2.1. Animals

Forty KM mice (20 ± 2 g) were provided by the Experimental Animal Center of Jilin University (Changchun, China). The animals were housed in normal cages under controlled environmental conditions (25°C and a 12 h light/dark cycle) and allowed to have free access to standard pellet diet (SPD) and water ad libitum. Experiments were performed in accordance with the Guide for the Care and Use of Laboratory Animals of Jilin University and approved by the ethics committee (approval number: 20180046).

### 2.2. Experimental Design

The mice were randomly divided into the following study groups: the normal control group (Control) was treated with double distilled water (20 mL/kg); the model control group (Model) was treated with diphenoxylate (Lot: 20180413, Changchun Changhong Pharmaceutical Co., Ltd, Changchun, China) (10 mg/kg); the low-dose YTC group (Lot: 20171205, Jilin Connell Pharmaceutical Co., Ltd, Jilin, China) (L-YTC) was treated with diphenoxylate (10 mg/kg) and 0.6 g/kg YTC; the high-dose YTC group (H-YTC) was treated with diphenoxylate (10 mg/kg) and 1.2 g/kg YTC. Diphenoxylate (0.025 mg atropine and 2.5 mg diphenoxylate per tablet) was used to induce constipation in the mice, as previously reported [28, 29]. Diphenoxylate was administered once daily for 14 consecutive days, whereas YTC was administered once daily in days 15–28 in the L-YTC and H-YTC groups. All drugs were administered via gastric gavage.

### 2.3. Fecal Parameter Measurement

After the administration of the drug on the final day, each group of mice was given 10% charcoal and placed in metabolic cage individually for 6 h. The discharge time of the first black stool and the weight of the feces within 6 h were recorded. Then, the percentage of water in the feces was calculated as follows: (wet weight of feces − dry weight of feces)/wet weight of feces × 100%.

### 2.4. Small Intestine Movement Trial

After 14 days of treatment, all mice were subjected to 12 h fasting but were allowed free access to water. Then, mice in each group were given 10% charcoal (20 mL/kg), and 30 minutes later, the small intestine was quickly removed and the total length of the small intestine (the distance from the pylorus to the ileocecal area) and the distance traveled by the charcoal were measured. The charcoal propulsion rate was calculated as charcoal propulsion distance/total small intestine length × 100%.

### 2.5. Blood Sample and Tissue Collection

At the end of the experimental period, mice were anesthetized 30 minutes later by intraperitoneal injection of sodium pentobarbital and placed on a temperature-regulated table. Blood samples were collected and centrifuged at 2500 rpm for 10 min to obtain serum. The colon was split immediately and flushed with saline solution at 4°C and then divided into two pieces. One fragment was fixed in 10% formalin and processed in split paraffin for subsequent histological analysis and immunohistochemical (IHC) analysis, while the other was stored at −80°C until they were assayed.

### 2.6. Histological Analysis

The colon was fixed in 4% formaldehyde, rinsed with running water for 24 h, and dehydrated with gradient ethanol (30%, 50%, 80%, 95%, 95%, 100%, and 100%). Then, it was wax-embedded and then sectioned into 5 *μ*m thick slices and oven-baked at 60°C for later use. Then, through xylene dewaxing, gradient alcohol hydration, hematoxylin and eosin staining, ascending gradient alcohol dehydration, xylene transparent, neutral gum seal, and other operations, pathological sections of colon tissue were prepared. Pathological changes in the colon of the mice were observed under a light microscope (200x).

### 2.7. Assessment of MTL, GT, SST, SP, NO, and Ach

The concentrations of MTL (Lot: I6RCWADD, Elabscience Biotechnology Co., Ltd, Wuhan, China), GT (Lot: Q9EYWD9X, Elabscience Biotechnology Co., Ltd, Wuhan, China), SST (Lot: UU41CID3, Elabscience Biotechnology Co., Ltd, Wuhan, China), and SP (Lot: FMSSCCLY, Elabscience Biotechnology Co., Ltd, Wuhan, China) in serum were estimated by ELISA using commercially available kits. While the levels of NO (Lot: 20180614, Nanjing Jiancheng Bioengineering Institute, Nanjing, China) and Ach (Lot: 20180704, Nanjing Jiancheng Bioengineering Institute, Nanjing, China) were determined by using diagnostic kits according to the manufacturer instructions.

### 2.8. Immunohistochemical Analysis

Wax-embedded colon tissues were cut into 5 *μ*m thick slices. After dewaxed by xylene, tissues were dehydrated with gradient ethanol and soaked in 3% H_2_O_2_ to remove endogenous peroxidase and subjected to antigen repair for 15 min. Then tissues were incubated with primary antibodies(1:100) overnight at 4 ℃ and incubated with a secondary antibody for 1h and streptavidin-peroxidase for 30 min at room temperature. The sections were observed under an optical microscope (200x). Determination of optical density (OD) values was done using Motic Images Advanced 3.2 image analysis software.

### 2.9. Western Blot Analysis

Colon tissue samples were homogenized and lysed in SDS-PAGE sample buffer. The protein concentration was determined using the BCA protein assay kit (Lot: B68010, Yeasen Biotech Co., Ltd, Shanghai, China). Then, the protein was boiled and centrifuged at 12,000 rpm for 15 min, after which the supernatant was collected. Equal amounts of protein from each sample were separated on 10% SDS-PAGE, transferred onto PVDF membranes, and incubated with 5% bovine serum albumin for 1 h. The PVDF membranes were probed with P2X2 (Lot: AD042895, Cell Signaling Technology, Inc., Danvers, MA, USA), C-kit (Lot: AE090855, Cell Signaling Technology, Inc., Danvers, MA, USA), and SCF (Lot: AD051429, Cell Signaling Technology, Inc., Danvers, MA, USA) antibody at 4°C overnight. Next, the PVDF membranes (Bio-Rad Laboratories, Hercules, CA, USA) were washed and incubated with horseradish-peroxidase-linked secondary antibodies. Changes in the density of the protein bands were quantified with ImageJ software.

### 2.10. Statistical Analysis

The results are presented as means ± SD. Data were analyzed by Student's *t*-test. GraphPad Prism 7.0 software was used for statistical analysis. *P* < 0.05 was considered statistically significant.

## 3. Results

### 3.1. Effect of YTC on Stool Number and Moisture Content in Diphenoxylate-Induced Constipation Mice

**Figure 1:**
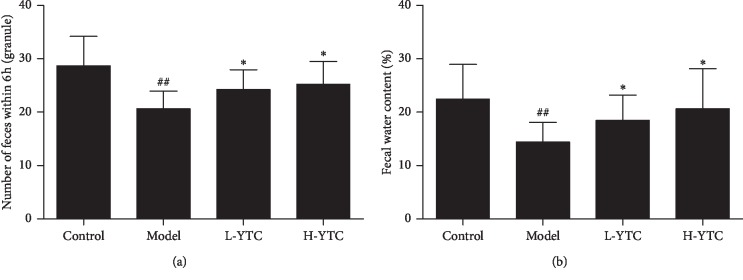
YTC improved stool number and moisture content in diphenoxylate-induced constipation mice. Analysis of (a) number of feces within 6 h and (b) fecal water content within 6 h, ^##^*P* < 0.01 compared to the control group, ^*∗*^*P* < 0.05 compared to the model group, *n* = 10 per group.

Compared with the control group, the weight of feces and the total water content within 6 h in the model group were significantly decreased (*P* < 0.01). But when treated by YTC, the weight of feces and water content in the L-YTC group and H-YTC group increased significantly (*P* < 0.05) (Figure 1).

### 3.2. Effect of YTC on First Black Excretion Time and Intestinal Transit Rate in Diphenoxylate-Induced Constipation Mice

**Figure 2:**
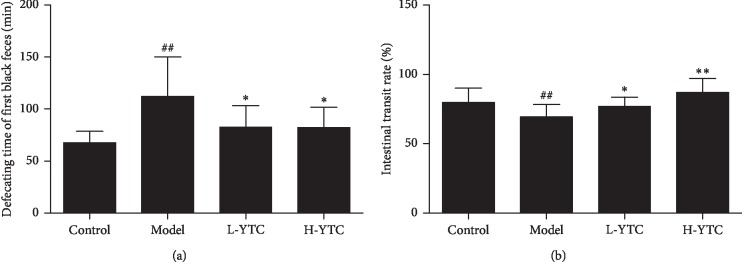
YTC shorten the defecating time of first black feces and improved the carbon propulsion rate in diphenoxylate-induced constipation mice. (a) Mice were given carbon by ig, and the defecating time of the first black feces was recorded. (b) Mice were given carbon by ig and then executed 30 minutes later. The small intestine was taken out and the propulsive rate of carbon was calculated. ^##^*P* < 0.01 compared to the control group, ^*∗*^*P* < 0.05 or ^*∗∗*^*P* < 0.01 compared to the model group, *n* = 10 per group.

The data showed that the first black excretion time in constipation mice was significantly prolonged, and the intestinal transit ratio decreased significantly in the model group compared with the control group (*P* < 0.01). However, the first black excretion time was significantly shortened whereas the intestinal transit ratio was significantly increased (*P* < 0.05 or *P* < 0.01) (Figure 2).

### 3.3. Histological Alterations of Colon

**Figure 3:**
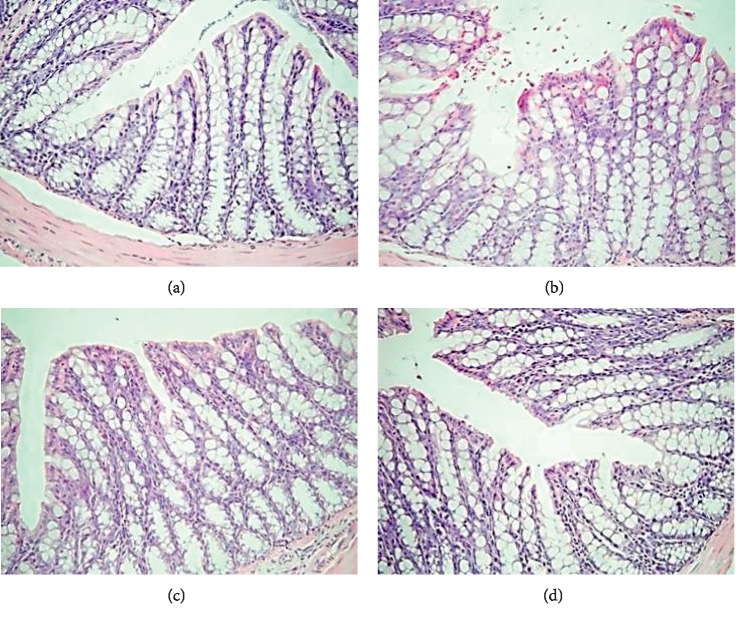
YTC attenuated histopathological changes in diphenoxylate-induced constipation mice. Representative H&E staining images of mice, 200x. *n* = 4 per group. (a) Control. (b) Model. (c) L-YTC. (d) H-YTC.

The histological alterations following treatment with YTC were investigated in the colons of mice with diphenoxylate-induced constipation by H&E staining. As shown in Figure 3, the mice treated with diphenoxylate alone exhibited a marked loss of epithelium of the colon and goblet cell depletion compared with the control group. Following treatment with YTC, moderately destructed epithelial cells were found when compared with the model group. The colons of mice in the H-YTC group exhibited intact epithelial cells and goblet cells, which were comparable to those of the control group.

### 3.4. Parameters of Serum

**Figure 4:**
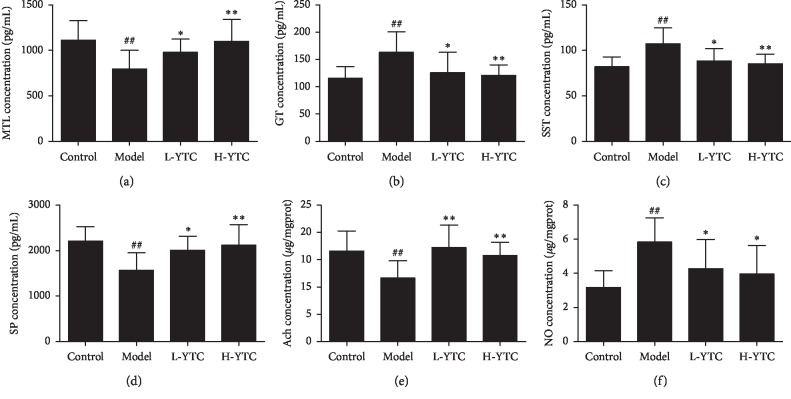
YTC regulates intestinal hormones and neurotransmitters in diphenoxylate-induced constipation mice. Contents of (a) MTL, (b) GT, (c) SST, and (d) SP in serum and (e) Ach and (f) NO in the colon were determined by diagnostic kits. ^##^*P* < 0.01 compared to the control group, ^*∗*^*P* < 0.05 or ^*∗∗*^*P* < 0.01 compared to the model group, *n* = 10 per group.

To evaluate the effects of YTC on serum biochemical components in the constipated mice, alterations of several components related to gastrointestinal metabolites in serum were assessed by ELISA and diagnostic kits. As shown in Figure 4, the levels of MTL, SP, and Ach were significantly decreased in the model group compared with the control group (*P* < 0.01) (Figures 4(a), 4(d), and 4(e)), while the levels of GT, SST, and NO were significantly increased (*P* < 0.01) (Figures 4(b), 4(c), and 4(f)). However, there is a reverse trend with these parameters after treatment with YTC compared with the model group (*P* < 0.05 or *P* < 0.01). Therefore, these results suggest that YTC treatment may regulate the factors related to gastrointestinal movement to relieve diphenoxylate-induced constipation in mice.

### 3.5. Effect of YTC on the Expression of AQP3 and AQP8 in Diphenoxylate-Induced Constipation Mice

**Figure 5:**
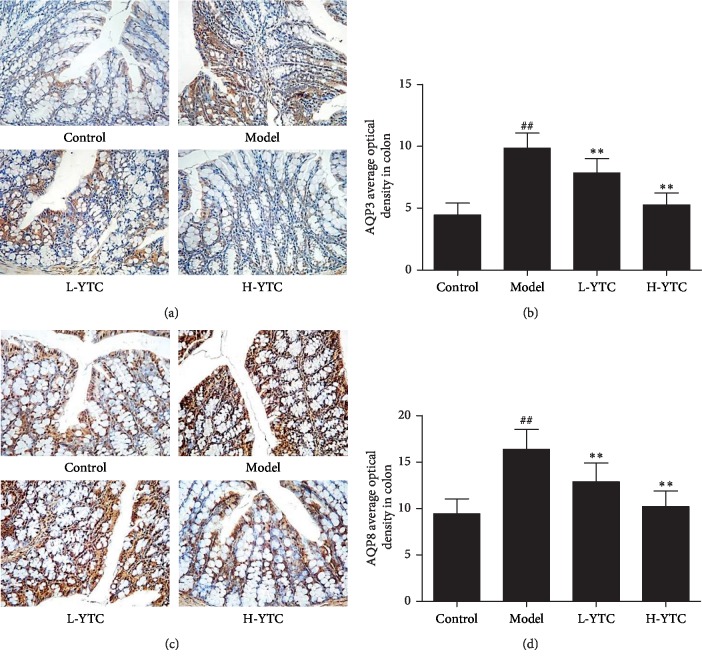
YTC reduced the expression of AQP3 and AQP8 in colon. (a) Protein expression of AQP3 in the colon was detected by immunohistochemistry. (b) The quantitative analysis of AQP3 was calculated. (c) Protein expression of AQP8 in the colon was detected by immunohistochemistry. (d) The quantitative analysis of AQP8 was calculated. ^##^*P* < 0.01 compared to the control group, ^*∗∗*^*P* < 0.01 compared to the model group, *n* = 4 per group.

AQP3 and AQP8 play an important role in regulating water transfer in the colon. Thus, we designed to examine the effect of YTC on the expression of AQP3 and AQP8 in the colons of mice with diphenoxylate-induced constipation. The expression levels of AQP3 and AQP8 in colon tissues were examined by IHC, and the quantitative analysis of OD was calculated. As shown in Figure 5, compared with the control group, diphenoxylate increased the expression levels of AQP3 and AQP8 (*P* < 0.01), whereas YTC decreased them (*P* < 0.01). The results suggest that YTC decreases the expression of AQP3 and AQP8 in the colons of mice with diphenoxylate-induced constipation.

### 3.6. Effect of YTC on the Expression of P2X2, SCF, and C-Kit in Diphenoxylate-Induced Constipation Mice

The P2X2 is a receptor which has been studied in recent years and belongs to the ATP-gated ion channel receptor [30]. The presence of P2X2 receptors in motor neurons and sensory neurons in the enteric nervous system suggests that it may play an important role in gastrointestinal motility and physiological functions of gastrointestinal sensation.

**Figure 6:**
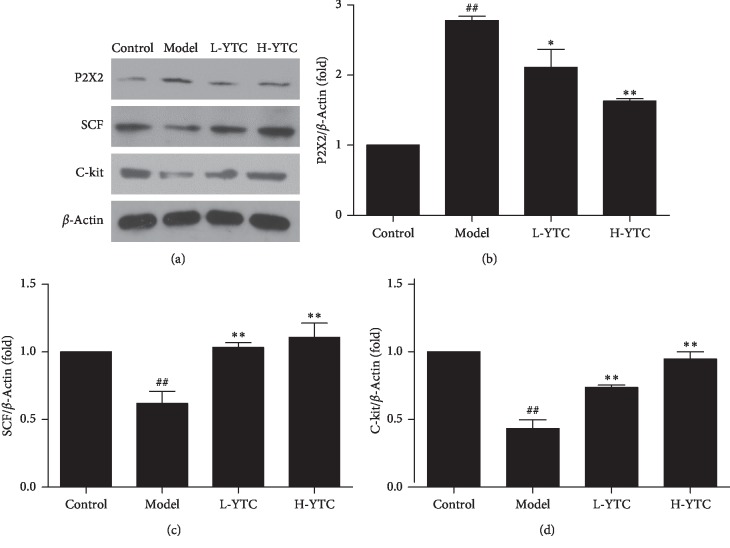
Protein expression of P2X2, SCF, and C-kit in the colon was detected by western blot. (a) Representative western blotting bands of P2X2, SCF, and C-kit. Semiquantitative analysis of (b) P2X2, (c) SCF, and (d) C-kit in each group. ^##^*P* < 0.01 compared to the control group, ^*∗*^*P* < 0.05 or ^*∗∗*^*P* < 0.01 compared to the model group, *n* = 3 per group.

While ICCs mainly distributed in the gastrointestinal tract, the main function is to regulate the gastrointestinal tract rhythmic movement, produce slow waves, and conduct electrical activity and participating in neurotransmission signal transduction. C-kit is recognized to be the marker of ICC with its receptor SCF that binds to it. In this study, to investigate the effects of YTC on P2X2 and ICC, the protein expression levels of P2X2, C-kit, and SCF in colon were determined by western blot analysis. The quantitative value of gray intensity analysis revealed that treatment with YTC decreased the expression level of P2X2, while C-kit and SCF increased (*P* < 0.05 or *P* < 0.01) (Figure 6). Therefore, these results suggest that constipation may be related to the expression of P2X2, C-kit, and SCF in the colon of mice.

## 4. Discussion

Constipation is one of the most common digestive tract diseases in clinic [31]. Its main symptoms are prolonged defecation cycle, fewer numbers of feces, and disappearance of defecation intention [32]. At the same time, abdominal distension, mental fatigue, reduced body mass, dark complexion, and other symptoms such as irritability, bad breath, and loss of appetite will occur in some patients [33]. YTC, which composed of herbal plants, has recently received increased attention as therapeutic drugs for the treatment of constipation, and our study suggests that YTC has a mitigative effect on mice with diphenoxylate-induced constipation.

Diphenoxylate acts directly on intestinal smooth muscle. It weakens peristalsis by inhibiting intestinal mucosal receptors and eliminating the peristaltic reflex of local mucosa [29]. At the same time, it can increase the segmental contraction of the intestine, thus prolonging the contact between intestinal contents and intestinal mucosa and promoting the absorption of water in the intestine. The results of this experiment showed that the intestinal transit function of mice decreased slightly, and there was no death in mice during the process of the experiment. It proves that diphenoxylate can successfully duplicate the model of constipation which was simple and safe.

Slow transit of colonic contents caused by colonic motility disorder is one of the main causes of constipation. Slow colonic movement, the decrease of fecal quantity, and water content are the main manifestations of constipation patients [34]. In this study, the effects of YTC on intestinal motility were evaluated by observing the fecal number, water content, the discharge time of first black stool, and the intestinal propulsion rate of constipated mice. Our study suggests that the intestinal propulsion rate, the discharge time of first black stool, amount of defecation, and wet weight of feces have significantly changed in the mice constipation model, while YTC can improve those parameters.

Abnormal expression of intestinal neurotransmitters may be the main pathogenesis of constipation. There are dozens of known intestinal neurotransmitters, including excitatory, inhibitory, and bidirectional neurotransmitters. Excitatory transmitters mainly include SP, Ach, etc. Inhibitory transmitters mainly include VIP, NO, and so on. When the intestinal nervous system functions normally, the neurotransmitters are in equilibrium. But when the balance between excitatory and inhibitory neurotransmitters is breaking, the intestinal neuromuscular dysfunction may be caused and affect defecation [35–37]. A variety of gastrointestinal hormones secreted by endocrine cells of the digestive tract also participate in the regulation of colonic motility [38, 39] such as MTL, GT, and cholecystokinin (CCK) which can promote intestinal motility, while SST inhibits intestinal motility. In the present study, compared with the model group, YTC was able to significantly increase the levels of MTL, SP, and Ach, while the levels of GT, SST, and NO decreased.

Aquaporins mainly mediate the passive transport of free water across biofilm, which plays an important role in maintaining the homeostasis of the intracellular and extracellular environment, and it also participates in some important physiological functions of the body [10]. Studies have shown that AQPs are widely distributed in various systems and play an indispensable role in the absorption and secretion of water [40]. AQP3 and AQP8 are closely related to the excessive absorption of water by colon and the decrease of intestinal secretion [41, 42]. Many gastrointestinal diseases such as diarrhea, gastritis, constipation, gastric cancer, and other diseases occur on account of the disorder of the dynamic balance of gastrointestinal water metabolism. Therefore, the abnormal expression of AQPs in the gastrointestinal tract is closely related to the occurrence of diseases. In this study, the expression level of AQP3 and AQP8 was detected and the results showed that YTC decreases the expression of AQP3 and AQP8 in the colons of mice with diphenoxylate-induced constipation.

ICC is a gastrointestinal pacemaker cell. It has the functions of producing slow wave, conducting slow-wave potential, regulating neurotransmitters, and so on [43, 44]. It is an important part of regulating gastrointestinal motility. The discovery of the tyrosine kinase receptor, C-kit, and its ligand, SCF, is critical in the development, maturation, and maintenance of the phenotype of ICC, which can be reliably identified by C-kit immunohistochemical techniques [21, 45, 46]. C-kit labeling indirectly reflects the quantity and density of ICC [47, 48]. It can not only maintain the phenotype of ICC, but also determine the differentiation, development, and rhythmic activity of ICC [49, 50]. Our results indicate that YTC increased the expression level of C-kit and SCF, which protect the expression of ICC.

## 5. Conclusion

In conclusion, our study found that YTC could alleviate constipation in mice. The underlying mechanisms might associate with its regulation on the content of intestinal hormones and neurotransmitters. In the meantime, YTC improved the slow-wave production of colon and regulated the contraction rhythm of smooth muscle by increasing ICC quantity through the SCF/C-kit signal pathway. Although the results from this study provide evidence that YTC has laxative effect on the mice constipation model, we cannot exclude other possibilities. This study serves as the foundation to further investigate its chemical components for the development of new drugs to better manage the disease and clarify other potential mechanisms.

## Data Availability

The data used to support the findings of this study are included within the article.

## References

[1] Hayat U., Dugum M., Garg S. (2017). Chronic constipation: update on management. *Cleveland Clinic Journal of Medicine*.

[2] Krogh K., Chiarioni G., Whitehead W. (2017). Management of chronic constipation in adults. *United European Gastroenterology Journal*.

[3] Payne I., Grimm L. (2016). Functional disorders of constipation: paradoxical puborectalis contraction and increased perineal descent. *Clinics in Colon and Rectal Surgery*.

[4] Woodward S. (2012). Assessment and management of constipation in older people. *Nursing Older People*.

[5] Mugie S. M., Di Lorenzo C., Benninga M. A. (2011). Constipation in childhood. *Nature Reviews Gastroenterology & Hepatology*.

[6] Mollen R. M. H. G., Hopman W. P. M., Kuijpers H. H. C., Jansen J. B. M. J. (2000). Plasma cholecystokinin, plasma peptide YY and gallbladder motility in patients with slow transit constipation: effect of intestinal stimulation. *Digestion*.

[7] Zhao R. H., Baig M. K., Thaler K. J. (2003). Reduced expression of serotonin receptor(s) in the left colon of patients with colonic inertia. *Diseases of the Colon & Rectum*.

[8] Björnsson E. S., Chey W. D., Hooper F., Woods M. L., Owyang C., Hasler W. L. (2002). Impaired gastrocolonic response and peristaltic reflex in slow-transit constipation: role of 5-HT3 pathways. *American Journal of Physiology-Gastrointestinal and Liver Physiology*.

[9] Bassotti G., Villanacci V., Maurer C. A. (2006). The role of glial cells and apoptosis of enteric neurones in the neuropathology of intractable slow transit constipation. *Gut*.

[10] Matsuzaki T., Tajika Y., Ablimit A. (2004). Aquaporins in the digestive system. *Medical Electron Microscopy*.

[11] Iantorno G., Bassotti G., Kogan Z. (2007). The enteric nervous system in chagasic and idiopathic megacolon. *The American Journal of Surgical Pathology*.

[12] Wood J. D. (2007). Effects of bacteria on the enteric nervous system. *Journal of Clinical Gastroenterology*.

[13] Yi R., Peng P., Zhang J. (2019). Lactobacillus plantarum CQPC02-fermented soybean milk improves loperamide-induced constipation in mice. *Journal of Medicinal Food*.

[14] Wattchow D., Brookes S., Murphy E., Carbone S., de fontgalland D., Costa M. (2008). Regional variation in the neurochemical coding of the myenteric plexus of the human colon and changes in patients with slow transit constipation. *Neurogastroenterology & Motility*.

[15] Pellegrini C., Fornai M., Colucci R. (2016). Alteration of colonic excitatory tachykininergic motility and enteric inflammation following dopaminergic nigrostriatal neurodegeneration. *Journal of Neuroinflammation*.

[16] Zhuang Z., Chen M., Niu J. (2019). The manufacturing process of Kiwifruit Fruit powder with high dietary fiber and its laxative effect. *Molecules*.

[17] Ikarashi N., Kon R., Sugiyama K. (2016). Aquaporins in the colon as a new therapeutic target in diarrhea and constipation. *International Journal of Molecular Sciences*.

[18] Sanders K. M., Koh S. D., Ward S. M. (2006). Interstitial cells of Cajal as pacemakers in the gastrointestinal tract. *Annual Review of Physiology*.

[19] Forrest A., Huizinga J. D., Wang X.-Y. (2007). Increase in stretch-induced rhythmic motor activity in the diabetic rat colon is associated with loss of ICC of the submuscular plexus. *American Journal of Physiology*.

[20] Wang X.-Y., Huizinga J. D., Diamond J., Liu L. W. C. (2009). Loss of intramuscular and submuscular interstitial cells of Cajal and associated enteric nerves is related to decreased gastric emptying in streptozotocin-induced diabetes. *Neurogastroenterology & Motility*.

[21] Farrugia G. (2008). Interstitial cells of Cajal in health and disease. *Neurogastroenterology & Motility*.

[22] Han J., Shen W.-H., Jiang Y.-Z. (2010). Distribution, development and proliferation of interstitial cells of Cajal in murine colon: an immunohistochemical study from neonatal to adult life. *Histochemistry and Cell Biology*.

[23] Li X., Liu Y., Guan W. (2019). Physicochemical properties and laxative effects of polysaccharides from *Anemarrhena asphodeloides* Bge. in loperamide-induced rats. *Journal of Ethnopharmacology*.

[24] Sharifi-Rad J., Melgar-Lalanne G., Hernández-Álvarez A. J. (2019). Malva species: insights on its chemical composition towards pharmacological applications. *Phytotherapy Research*.

[25] Cirillo C., Capasso R. (2015). Constipation and botanical medicines: an overview. *Phytotherapy Research*.

[26] Yingngam B., Zhao H., Baolin B. (2019). Optimization of ultrasonic-assisted extraction and purification of Rhein from Cassia fistula pod pulp. *Molecules*.

[27] Yan S., Yue Y.-Z., Wang X.-P. (2017). Aqueous extracts of *Herba Cistanche* promoted intestinal motility in loperamide-induced constipation rats by ameliorating the interstitial cells of Cajal. *Evidence-Based Complementary and Alternative Medicine*.

[28] Zhu F., Xu S., Zhang Y. (2016). Total glucosides of paeony promote intestinal motility in slow transit constipation rats through amelioration of interstitial cells of Cajal. *PLoS One*.

[29] Xu J., Zhou X., Chen C. (2012). Laxative effects of partially defatted flaxseed meal on normal and experimental constipated mice. *BMC Complementary and Alternative Medicine*.

[30] Axel S., Sylvia J., Ralf H. (2019). Bile acids are potent inhibitors of rat P2X2 receptors. *Purinergic Signalling*.

[31] Walia R., Mahajan L., Steffen R. (2009). Recent advances in chronic constipation. *Current Opinion in Pediatrics*.

[32] Udani J. K., Bloom D. W. (2013). Effects of kivia powder on Gut health in patients with occasional constipation: a randomized, double-blind, placebo-controlled study. *Nutrition Journal*.

[33] Mostafa S. M., Bhandari S., Ritchie G., Gratton N., Wenstone R. (2003). Constipation and its implications in the critically ill patient. *British Journal of Anaesthesia*.

[34] Moriya R., Fujikawa T., Ito J. (2010). Pancreatic polypeptide enhances colonic muscle contraction and fecal output through neuropeptide Y Y4 receptor in mice. *European Journal of Pharmacology*.

[35] Yik Y. I., Farmer P. J., King S. K., Chow C. W., Hutson J. M., Southwell B. R. (2011). Gender differences in reduced substance P (SP) in children with slow-transit constipation. *Pediatric Surgery International*.

[36] Peregud D. I., Yakovlev A. A., Stepanichev M. Y., Onufriev M. V., Panchenko L. F., Gulyaeva N. V. (2015). Expression of BDNF and TrkB phosphorylation in the rat frontal cortex during morphine withdrawal are NO dependent. *Cellular and Molecular Neurobiology*.

[37] Tomita R., Igarashi S., Fujisaki S. (2007). The effects of neurotensin in the colon of patients with slow transit constipation. *Hepatogastroenterology*.

[38] Liu Y., Zhao X. R., Wang R. (2008). Effect of Zhizhuwan on gastrointestinal peptide concentrations in plasma of diabetic gastroenteropathy with constipation patients. *Zhongguo Zhong Yao Za Zhi*.

[39] Suo H., Zhao X., Qian Y. (2014). Therapeutic effect of activated carbon-induced constipation mice with *Lactobacillus fermentum* Suo on treatment. *International Journal of Molecular Sciences*.

[40] Silberstein C., Kierbel A., Amodeo G. (1999). Functional characterization and localization of AQP3 in the human colon. *Brazilian Journal of Medical and Biological Research*.

[41] Kon R., Ikarashi N., Hayakawa A. (2015). Morphine-induced constipation develops with increased aquaporin-3 expression in the colon via increased serotonin secretion. *Toxicological Sciences*.

[42] Wang J.-P., Hou X.-H. (2007). Expression of aquaporin 8 in colonic epithelium with diarrhoea-predominant irritable bowel syndrome. *Chinese Medical Journal*.

[43] Xu J., Chen Y., Liu S. (2013). Electroacupuncture regulates apoptosis/proliferation of intramuscular interstitial cells of cajal and restores colonic motility in diabetic constipation rats. *Evidence-Based Complementary and Alternative Medicine*.

[44] Lees-Green R., Du P., O’Grady G. (2011). Biophysically based modeling of the interstitial cells of Cajal: current status and future perspectives. *Frontiers in Physiology*.

[45] Tan Y. Y., Ji Z. L., Zhao G. (2014). Decreased SCF/c-kit signaling pathway contributes to loss of interstitial cells of Cajal in gallstone disease. *International Journal of Clinical and Experimental Medicine*.

[46] Yu C., Kim H., Hong H. (2002). Evaluation of myenteric ganglion cells and interstitial cells of Cajal in patients with chronic idiopathic constipation. *International Journal of Colorectal Disease*.

[47] Mostafa R. M., Moustafa Y. M., Hamdy H. (2010). Interstitial cells of Cajal, the maestro in health and disease. *World Journal of Gastroenterology*.

[48] Parthasarathy G., Chen J., Chia N. (2017). Reproducibility of assessing fecal microbiota in chronic constipation. *Neurogastroenterology & Motility*.

[49] Horváth V. J., Vittal H., Lörincz A. (2006). Reduced stem cell factor links smooth myopathy and loss of interstitial cells of cajal in murine diabetic gastroparesis. *Gastroenterology*.

[50] Loera-Valencia R., Wang X. Y., Wright G. W. J. (2014). Ano1 is a better marker than c-Kit for transcript analysis of single interstitial cells of Cajal in culture. *Cellular & Molecular Biology Letters*.

